# Atypical manifestation of psoriasis in the elderly: possible expression of Mal de Meleda diagnosis review and case report

**DOI:** 10.3389/fmed.2025.1634843

**Published:** 2025-09-18

**Authors:** Cristian Onisor, Ana-Maria Sarpe, Elena Niculet

**Affiliations:** ^1^Faculty of Medicine and Pharmacy, “Dunarea de Jos” University of Galati, Galati, Romania; ^2^“Sf. Apostol Andrei” Emergency County Clinical Hospital Galati, Galati, Romania

**Keywords:** Mal de Meleda, palmoplantar keratoderma, psoriasis, PPK, IL-17A

## Abstract

Mal de Meleda (MDM) is a rare hereditary skin disorder classified as an autosomal recessive palmoplantar keratoderma, with an estimated prevalence of 1 in 100,000 individuals. It is commonly linked to consanguineous parentage and typically presents during early childhood. The hallmark features of the condition include transient thickening of the palms and soles, skin sclerosis and atrophy, scleroatrophic erythematous lesions, pseudoainhum formation around the digits, and erythema around the mouth. Due to its rarity, many dermatologists may lack clinical familiarity with MDM. Moreover, there is considerable phenotypic similarity between Mal de Meleda and other forms of palmoplantar keratoderma, as well as certain erythrokeratodermas, making clinical differentiation particularly difficult. Therefore, genetic testing to identify causative mutations plays a crucial role in confirming the diagnosis. Genetic alterations in the *SLURP1* gene, which encodes the secreted Ly-6/uPAR-related protein 1, have been identified as key contributors to the development of Mal de Meleda (MDM). SLURP1 plays a critical role in maintaining epidermal equilibrium by regulating essential processes such as keratinocyte (KC) proliferation, terminal differentiation, programmed cell death (apoptosis), and cornification. Furthermore, reduced SLURP1 expression has been linked to the pathogenesis of various epithelial cancers, suggesting its broader significance in maintaining epithelial tissue integrity and suppressing tumors. The management of Mal de Meleda remains a clinical challenge, as current therapeutic approaches are largely limited to alleviating symptoms rather than addressing the underlying cause. Systemic retinoids, particularly acitretin, have demonstrated efficacy in reducing epidermal hyperkeratosis and enhancing clinical outcomes. Nonetheless, prolonged administration may lead to significant side effects, necessitating close clinical supervision. This case report contributes to the medical literature by documenting a rare presentation of Mal de Meleda, highlighting the importance of recognizing atypical clinical features and implementing accurate diagnostic and therapeutic interventions. Psoriasis is a chronic, immune-mediated inflammatory dermatosis that exerts a profound effect on patients’ quality of life. The disease typically follows a relapsing-remitting course and is frequently associated with a spectrum of comorbid conditions. In pediatric populations, the etiology of psoriasis is multifactorial, involving a combination of genetic susceptibility, environmental triggers, psychosocial stressors, obesity, physical trauma, cutaneous irritation, and exposure to certain pharmacological agents such as lithium, β-blockers, and tumor necrosis factor (TNF) inhibitors. Moreover, systemic inflammatory disorders like Crohn’s disease and juvenile idiopathic arthritis have been linked to an increased risk of psoriasis in children. The disease burden is considerable in adults, but poses even greater challenges in pediatric patients, where atypical clinical presentations may hinder timely and accurate diagnosis. It is essential for dermatologists to accurately differentiate Mal de Meleda (MDM) from psoriasis, as these conditions may exhibit overlapping clinical features yet diverge significantly in therapeutic response and long-term prognosis. Distinctive diagnostic clues—such as very early disease onset, autosomal recessive mode of inheritance, and the hallmark transgradient pattern of palmoplantar keratoderma—can aid in distinguishing MDM from the more prevalent psoriasis vulgaris. Although biologic therapies targeting TNF-α and IL-17A have shown limited efficacy in MDM, systemic retinoids continue to represent a clinically effective therapeutic option for managing this rare genodermatosis.

## Introduction

Mal de Meleda (MDM) represents a rare subtype of palmoplantar keratoderma (PPK), inherited in an autosomal recessive pattern, with an estimated prevalence of approximately 1 in 100,000 individuals (1). The condition was initially documented by Stulli in 1826 and subsequently characterized in more detail by Meleda in 1829, from which its nomenclature is derived (2). Clinical manifestations of PPK typically arise between birth and the age of three years. Hallmark features include transgradient hyperkeratosis with well-defined borders, nail dystrophy, scleroatrophic erythematous lesions, the formation of pseudoainhum around the digits, and erythema localized to the perioral region (1).

The therapeutic management of Mal de Meleda remains complex, as current treatment modalities are largely palliative, aiming to relieve symptoms rather than cure the disease. Systemic retinoids, such as acitretin, have demonstrated clinical efficacy in mitigating hyperkeratosis and alleviating associated symptoms (3). Nevertheless, prolonged administration is frequently linked to potential adverse effects, necessitating vigilant monitoring and individualized risk-benefit assessment (4). This case report enhances the existing body of knowledge by detailing a rare presentation of Mal de Meleda, underscoring the need for heightened clinical awareness of atypical phenotypes and the adoption of precise diagnostic and therapeutic approaches.

Pathogenic variants in the *SLURP1* gene, which encodes the secreted mammalian Ly-6/uPAR-related protein-1 (SLURP1), have been identified as causative in the development of Mal de Meleda (MDM). SLURP1 plays a pivotal role in maintaining epidermal homeostasis by regulating key biological processes such as keratinocyte (KC) proliferation, terminal differentiation, programmed cell death (apoptosis), and cornification (5). Furthermore, diminished SLURP1 expression has been associated with the pathogenesis of several epithelial malignancies, indicating its broader relevance in epithelial tissue integrity and tumor suppression (6).

From a clinical standpoint, many dermatologists may lack familiarity with Mal de Meleda (MDM) due to its rarity. Moreover, there is considerable phenotypic overlap between MDM, certain erythrokeratodermas, and various forms of palmoplantar keratoderma, making accurate clinical differentiation particularly difficult. As such, reliance solely on physical examination and symptomatology is often insufficient, and genetic analysis to identify pathogenic mutations is essential for establishing a definitive diagnosis.

In contrast, psoriasis is a relatively common, chronic inflammatory dermatosis with a well-documented genetic predisposition, marked by intricate disturbances in epidermal proliferation and differentiation. Although pediatric psoriasis is increasingly recognized, its exact prevalence remains uncertain. This condition can profoundly affect a child’s life, impairing physical health, emotional wellbeing, and social interactions, thereby diminishing overall quality of life. Importantly, children should not be viewed as merely smaller versions of adults; pediatric psoriasis is distinct from adult-onset forms in terms of epidemiology, clinical features, therapeutic strategies, and its long-term medical and psychosocial consequences (7).

## Epidemiology

The majority of documented cases of Meleda disease have originated from regions in and around the former Yugoslavia. The estimated prevalence is approximately one in 100,000 individuals (1, 8). Clinical manifestations typically emerge shortly after birth, and the condition does not exhibit any predilection based on sex or ethnic background, indicating an equal susceptibility across populations (8).

**FIGURE 1:**
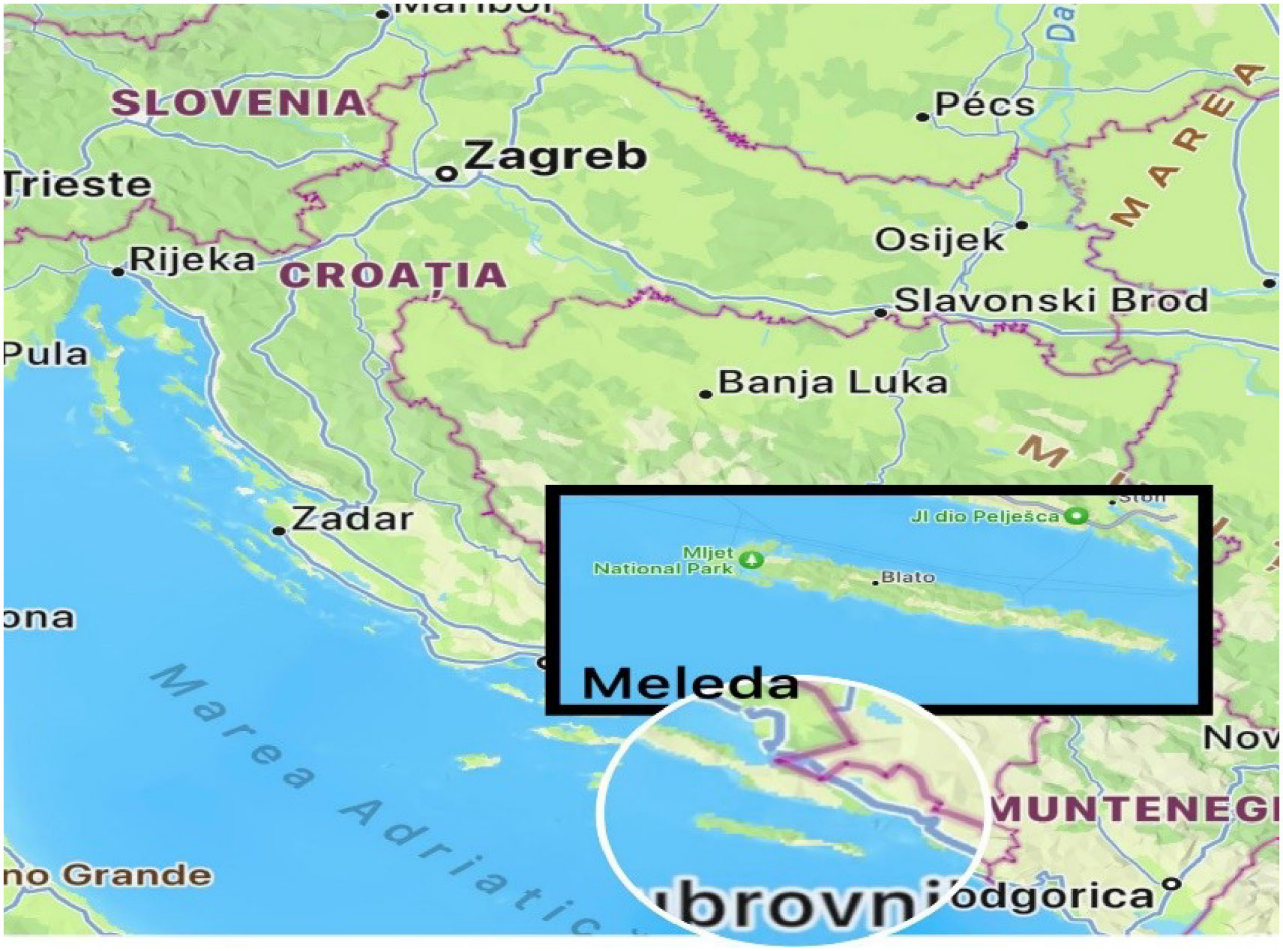
The geographical location of Meleda island in the Adriatic Sea, where the family described in this case originates.

It is hypothesized that Meleda disease originated on the Croatian island of Mljet (Figure 1), where individuals were isolated in 1826 due to outbreaks of plague and other infectious conditions (8). During this period of seclusion, consanguineous relationships are believed to have occurred, contributing to the emergence and propagation of Meleda disease within the local population (8).

Psoriasis is a chronic, immune-mediated inflammatory dermatosis. In the pediatric population, its prevalence is estimated to range from 0.5% to 8.5%, with certain studies indicating a slightly higher incidence among females (9–11).

### Materials and methods

Informal consent was obtained from the patient, and the research complies with the ethical principles of the Declaration of Helsinki. Approval was obtained from the Ethics Committee of Emergency Hospital “Sfantul Apostol Andrei” Galati, No.4654 on the date of 04.03.2025.

### Clinical case

We report the case of a 75-year-old patient from a rural area who presented to the Emergency Department with marked asthenia, wheezing, and mild dyspnea. The patient stated that he had been suffering from this condition since around the age of one. Family history revealed the presence of the same condition in another family member, namely the patient’s sister. However, his four children did not suffer from this disorder.

The patient had never undergone a dermatological consultation, so the diagnosis had not confirmed. His medical history includes type II diabetes mellitus treated with oral antidiabetic medications, diagnosed at the age of 71, and stage III arterial hypertension, for which treatment has been neglected.

**FIGURE 2:**
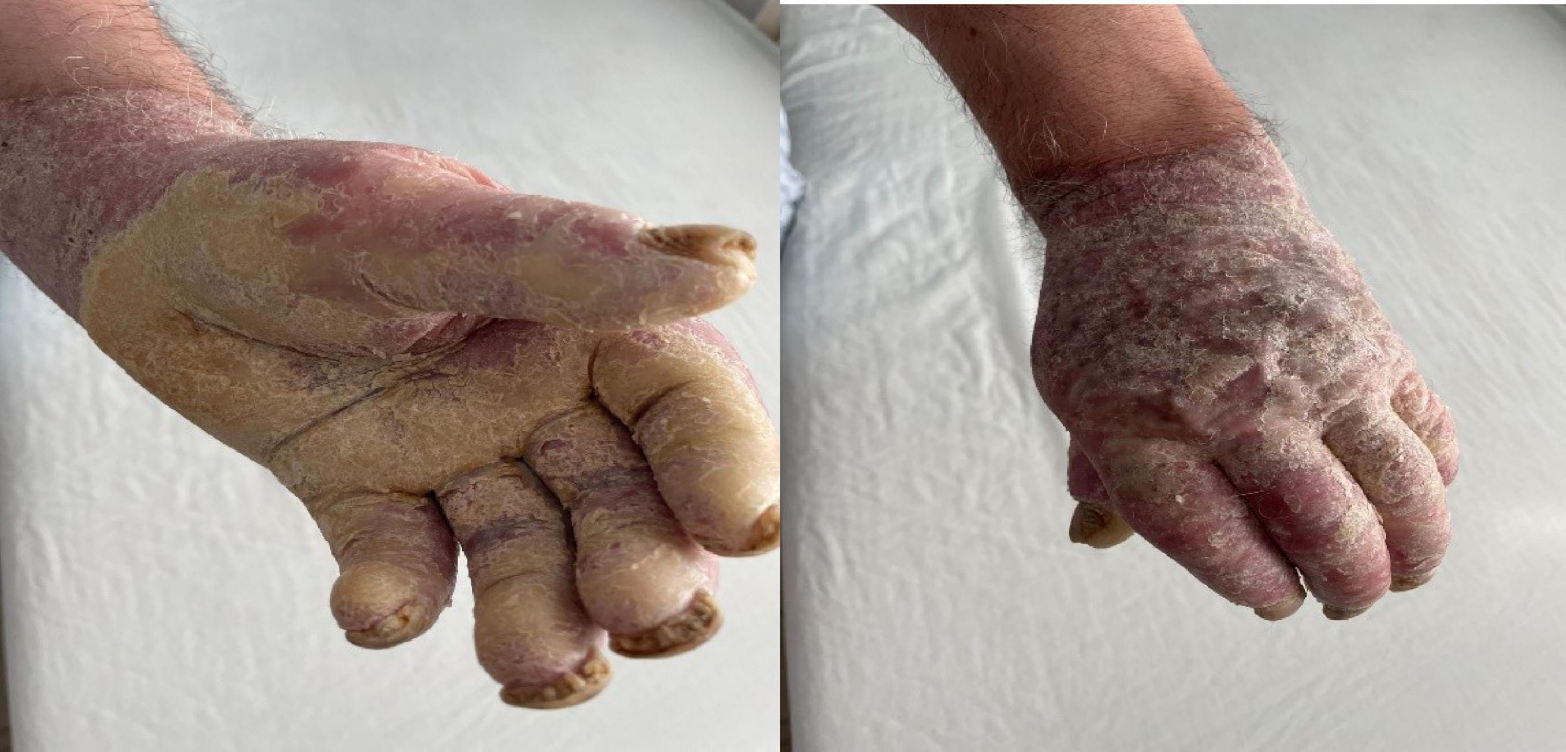
Extensive hyperkeratosis with fissures extended over the wrist and whitish scales.

**FIGURE 3:**
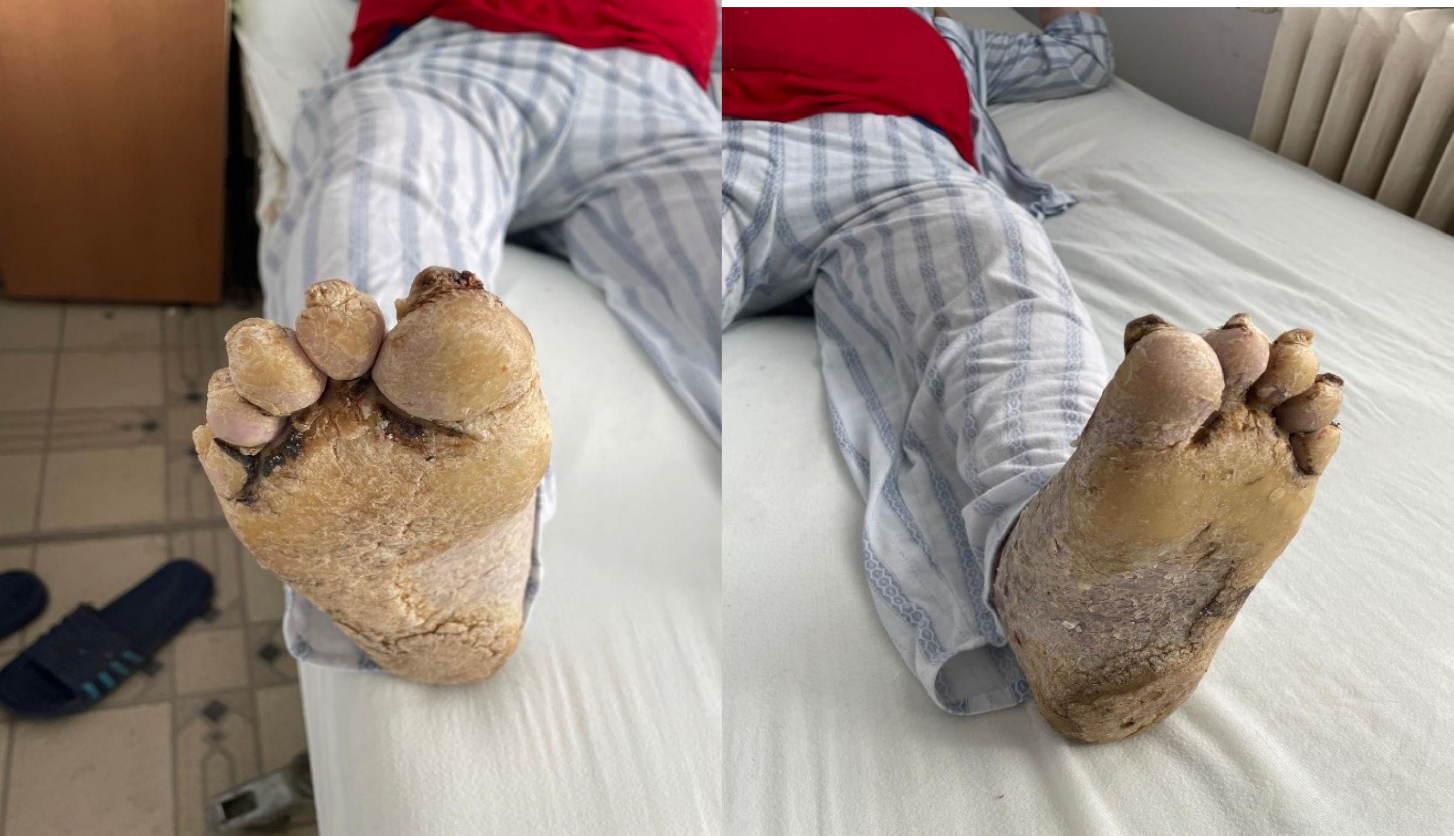
Extensive hyperkeratotic plaque with well demarcated erythema and onicodistrophy. Dorsal side of the foot presenting diffuse erythema, scaling, severe nail dystrophy and several pustular lesions on the halux.

**FIGURE 4:**
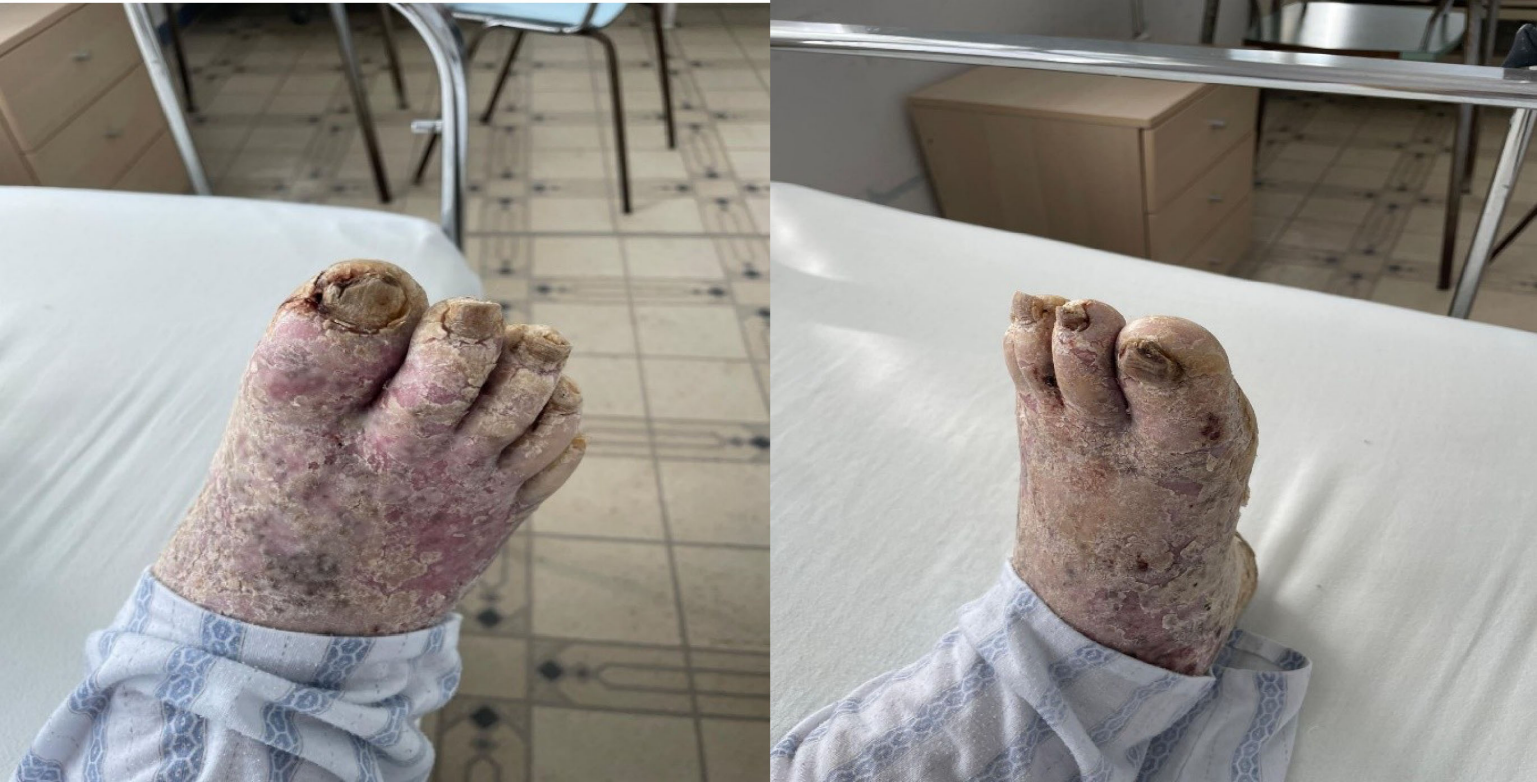
Plantar side of the foot affected by severe yellowish hyperkeratosis especially on the pressure areas. The nails are thickened with hyperkeratotic material underneath.

His occupational history reveals that the patient was a driver and resides in a rural area. General clinical examination shows a BMI of 61.98, emphysematous facies, and morbid obesity. The skin shows a dense, hyperkeratotic, waxy layer covering the entire surface of the palms and soles. The keratoderma extends proximally to the wrists and the soles of both feet. On the dorsal sides of the hands and feet, poorly defined erythematous-squamous plaques of varying sizes and shapes could be observed (Figures 2–4).

The patient stated that he had been suffering from this condition since around the age of one. Family history indicates that his parents and grandparents had no dermatological conditions. Among his siblings, he mentioned a sister who had similar lesions but passed away due to a cardiovascular condition. The patient also mentioned that he has four children (ages not specified), none of whom have dermatological lesions.

Although dermatological consultations had been recommended by his family doctor over the years, the patient did not avail himself of these services. This is his first hospital admission, during which dermatology, plastic surgery, and histopathological examinations were requested.

The nails show onychomycosis. Subcutaneous adipose tissue is excessively represented. The muscular system is normotonic and normokinetic. The osteoarticular system is clinically intact but shows arthralgia in the hands and feet.

Respiratory system examination: intermittent wheezing, kyphotic thorax, equal and symmetrical costal excursions, vocal fremitus is weakly transmitted on both hemithoraces, and respiratory rate at admission is 12 breaths/min.

Cardiovascular, digestive, renal, and neurological systems without pathological changes.

Paraclinical examination reveals mild leukocytosis (11.80 uL), hyperglycemia (414.44 mg/dl), moderate inflammatory syndrome (ESR = 40 mm/h, CRP = 37.20 mg/L), and negative tumor markers.

**FIGURE 5:**
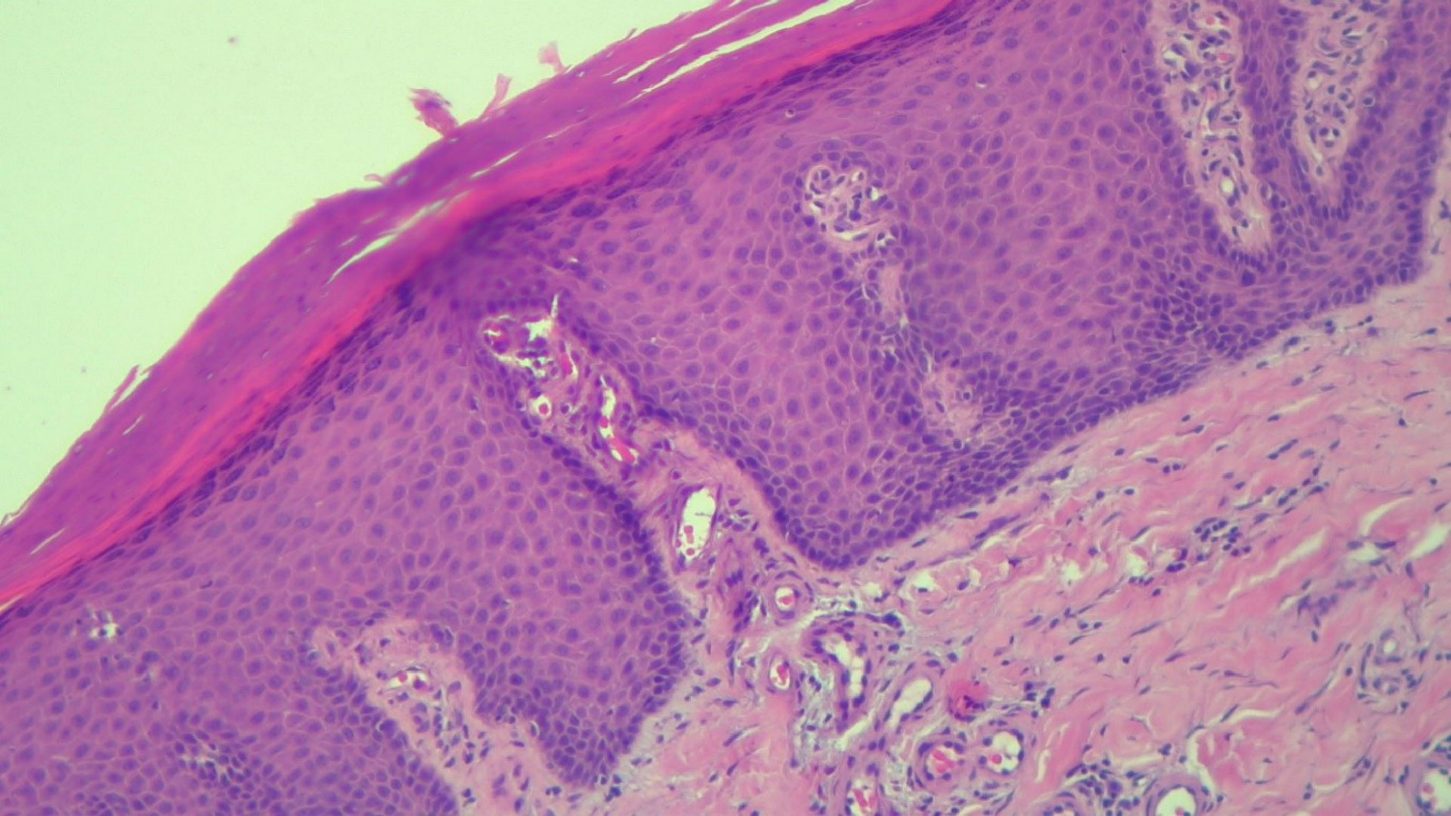
HEx100—Skin section showing marked hyper-parakeratosis with a granular layer of variable thickness, a regular, psoriasiform, acanthotic epidermis with tortuous capillaries in the superficial dermis and chronic inflammatory infiltrate.

**FIGURE 6:**
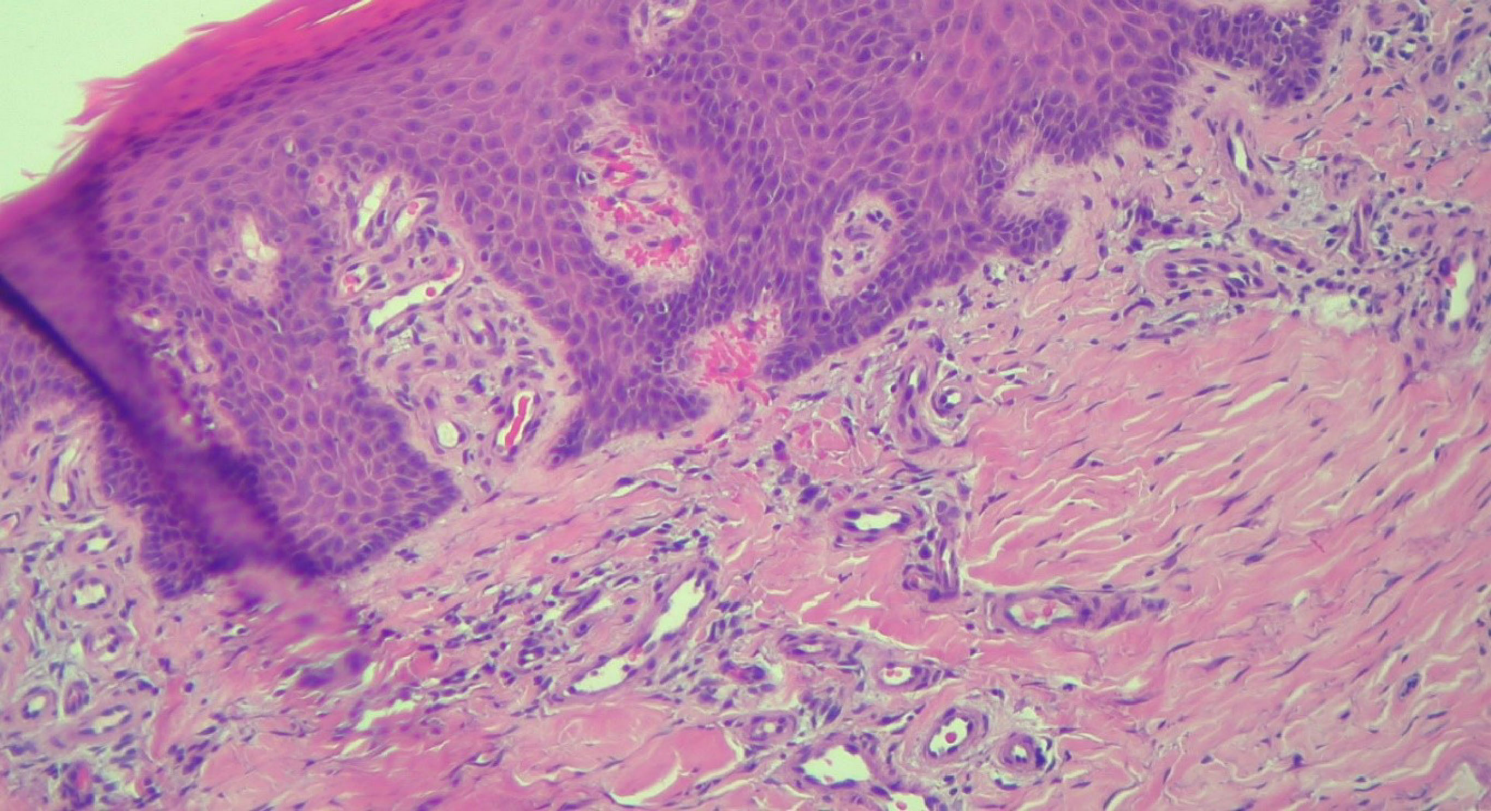
HEx100—Extreme tortuosity of dermal capillaries, extravasated erythrocytes.

**FIGURE 7:**
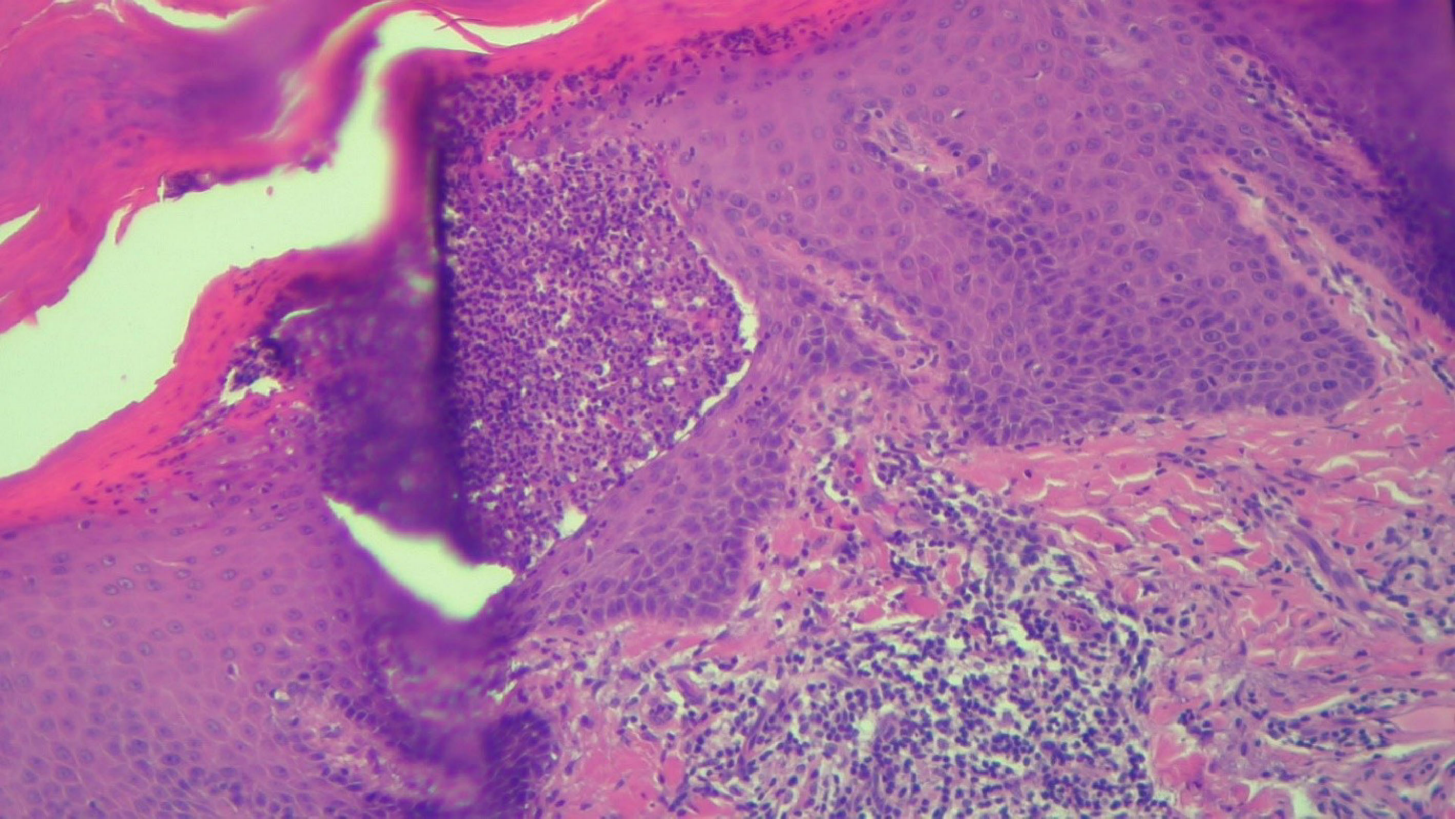
HEx100—Another skin biopsy aspect of the same patient with Kogoj pustule.

**FIGURE 8:**
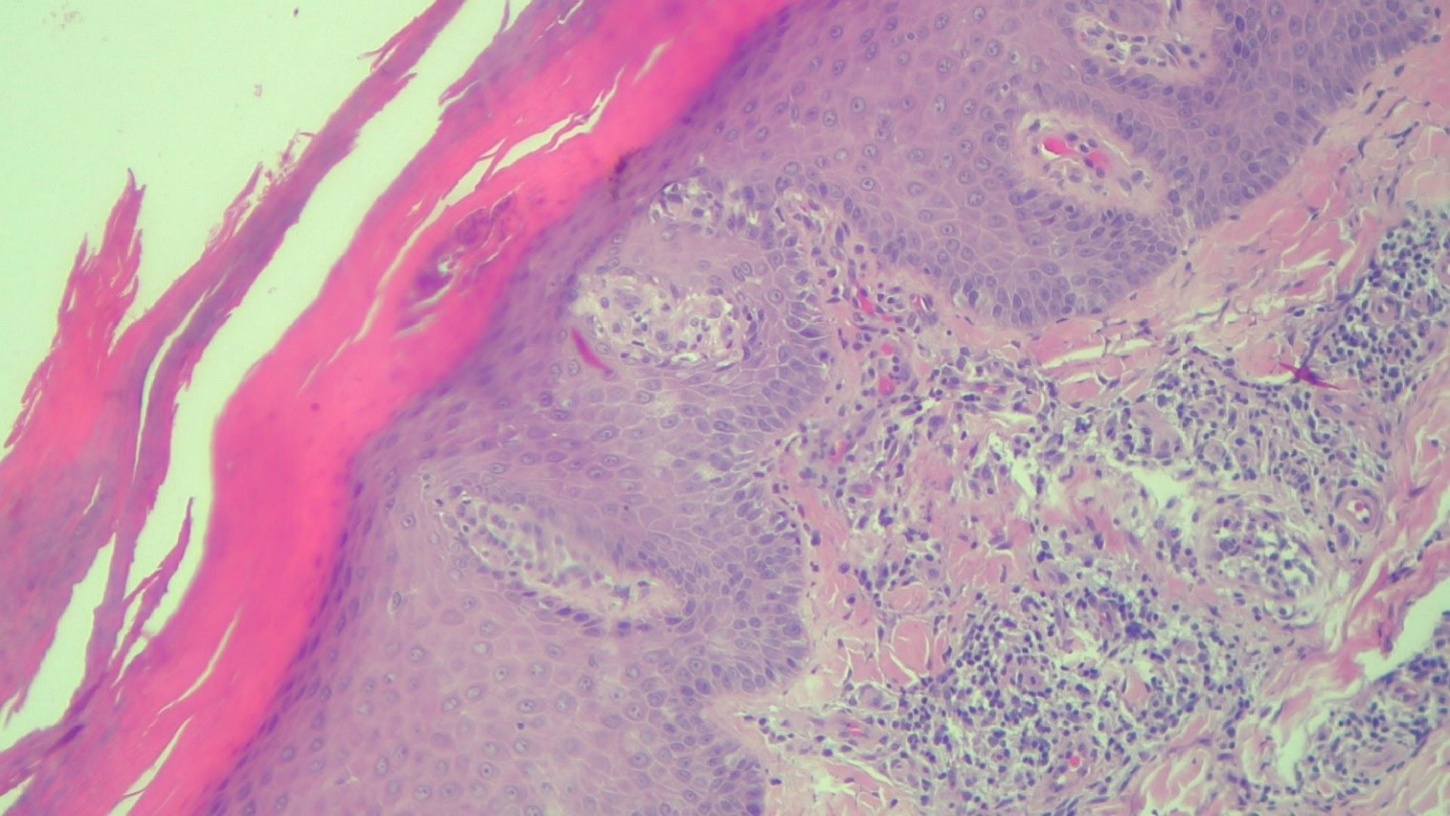
HEx100—Same patient with Munro microabcesses in the parakeratotic scale, focal agranulosis and more abundant inflammation in the superficial dermis.

The histopathological examination revealed (Figures 5–8). The examined skin fragment reveals an epidermis with marked hyperkeratosis, both ortho- and parakeratosis (confluent, with accumulations of neutrophils at this level—Munro microabscesses), a granular layer of variable thickness, with hypergranulosis and agranulosis, Kogoj pustules, regular psoriasiform acanthosis with elongated epidermal ridges, some rounded at the ends and fused at this level. The superficial dermis shows congested blood vessels, some with a tortuous appearance, and a perivascular inflammatory infiltrate consisting of lymphocytes, histiocytes, neutrophils (minimal), and extravasated red blood cells. Areas of erythrocyte exocytosis are also observed at the epidermal level.

The described histopathological features are consistent with a diagnosis of psoriasis vulgaris. Note: Considering the patient’s clinical presentation and history, Mal de Meleda can be considered as a differential diagnosis. A clinicopathological correlation is recommended, along with further examination using special stains for fungi (PAS), and referral to a dermatology clinic.

Singer et al. described a similar case (12). A seven-year-old girl with an erythematous-squamous skin rash was admitted to our clinic for diagnosis and treatment, following a transfer from a county hospital. Her family history included a maternal aunt with psoriasis. Upon admission, the patient exhibited prominent, well-defined, non-itchy erythematous plaques, covered with thick, silvery-white scales that were easily exfoliated. The lesions were present on the scalp, ear lobes, neck, trunk, limbs, periungual areas, as well as the axillary and genital regions—dermatological examination led to a diagnosis of psoriasis vulgaris in patches and plaques, which was confirmed histopathologically. With local dermatological treatment, the condition improved; the thick scales diminished, and gradual clearance of the psoriasis was observed. This case was highlighted due to the rarity of psoriasis vulgaris appearing at such an early age.

### Dermatological diseases and quality of life

Extensive research examining the impact of dermatological diseases on quality of life has provided valuable understanding in recent years. Individuals affected by these conditions—particularly those living with psoriasis—often experience considerable physical, emotional, and social distress (13–15). Findings by Mease and Menter (16) reveal that quality of life is significantly compromised in patients with widespread psoriasis or when lesions are located on visible or sensitive areas, including the face, hands, or genital region. Additionally, symptoms such as cutaneous pain and discomfort (17–19) have been shown to negatively affect essential aspects of daily functioning, including sleep quality, emotional wellbeing, and overall life satisfaction (20–22). Global studies further support a strong correlation between psoriasis and psychological disorders, especially depression and anxiety (23–26). Importantly, younger patients and those with severe clinical presentations appear to be at greater risk of experiencing these mental health challenges (27–29).

## Discussion

Mal de Meleda (MDM) is characterized by transgradient erythematous lesions primarily affecting the palms and soles, with cutaneous manifestations that frequently progress with advancing age. In some cases, MDM may mimic psoriasis, particularly when additional anatomical sites such as the scalp, elbows, or knees are involved. Accurate diagnosis of MDM is based on several key criteria, including its mode of inheritance, age of onset, morphological characteristics of lesions, and the specific pattern of hyperkeratosis distribution. Genetic testing remains the definitive method for diagnosis and plays a critical role in guiding therapeutic decisions (29).

Differentiating MDM from psoriasis is essential, given the variation in their responses to biologic agents and the elevated risk of skin malignancies associated with MDM. Typically, MDM presents shortly after birth and is inherited in an autosomal recessive manner. Although both conditions may exhibit well-demarcated erythematous plaques with scaling and nail abnormalities such as subungual hyperkeratosis and onycholysis, MDM is uniquely defined by its “stocking and glove” distribution pattern (transgradiens). Furthermore, features such as sclerodactyly and constrictive bands around the digits (pseudoainhum), which are prominent in MDM, are rarely observed in psoriasis.

From a histopathological perspective, differentiating between psoriasis and Mal de Meleda (MDM) can be challenging, as both may exhibit overlapping features such as parakeratosis, orthokeratosis, epidermal hyperplasia (acanthosis), Munro’s microabscesses, and a perivascular lymphocytic infiltrate. Nevertheless, immunohistochemical analysis reveals that SLURP-1 mRNA expression is upregulated by interleukin (IL)-22 in psoriatic lesions, whereas SLURP-1 is either absent or expressed at very low levels in MDM. Consequently, SLURP-1 immunostaining can serve as a useful diagnostic tool to distinguish between the two conditions.

The cytokine milieu involving TNF-alpha, IL-17, and IL-22 is well-established in the pathogenesis of psoriasis. However, the immunological pathways implicated in MDM remain insufficiently defined. Biologic therapies targeting TNF-alpha or IL-17A, which have shown efficacy in treating psoriasis, generally yield limited therapeutic benefit in MDM. Management of MDM typically involves systemic retinoids and topical keratolytic agents, whereas immunosuppressive drugs such as methotrexate and cyclosporine—commonly used in psoriasis—are largely ineffective in MDM cases.

Recent investigations indicate that pediatric patients with psoriasis have approximately double the risk of developing comorbid conditions compared to healthy children. Psoriasis has been associated with several autoimmune and inflammatory diseases, including type 1 diabetes, rheumatoid arthritis, Crohn’s disease, systemic lupus erythematosus, vitiligo, alopecia areata, eczema, and lichen planus. While psoriasis can be clinically mistaken for seborrheic dermatitis or nutritional deficiencies such as avitaminosis, these conditions may also coexist with psoriasis. In pediatric populations, psoriasis is frequently observed alongside atopic dermatitis.

Furthermore, childhood psoriasis—particularly in boys—is associated with a higher prevalence of obesity, which correlates with increased disease severity compared to children of normal body weight. One of the most critical associated conditions is psoriatic arthritis, although its exact prevalence among children remains unclear. Psoriatic arthritis can manifest at any age but commonly presents between 2–3 and 9–12 years. It typically causes joint discomfort, predominantly in the digits. Younger children, especially girls, more frequently exhibit oligoarticular involvement or dactylitis, whereas older boys often demonstrate features of enthesitis and axial skeletal involvement.

Clinically, a considerable number of dermatologists may lack familiarity with this uncommon genetic disorder. Moreover, Mal de Meleda (MDM) shares phenotypic characteristics with erythrokeratodermas and various forms of palmoplantar keratoderma, which complicates the diagnostic process. As a result, relying solely on clinical presentation often proves insufficient for an accurate diagnosis. Molecular genetic testing to identify pathogenic mutations plays a pivotal role in confirming the diagnosis of MDM.

Singer et al. reported a similar case involving a seven-year-old girl who presented with an erythematous-squamous skin rash. She was referred to our clinic for diagnosis and treatment after being transferred from a county hospital. Her family history revealed a maternal aunt with psoriasis. Upon examination, the patient displayed prominent, well-defined, non-itchy erythematous plaques covered with thick, silvery-white scales that were easily exfoliated. The lesions were distributed across the scalp, ear lobes, neck, trunk, limbs, periungual areas, as well as the axillary and genital regions. A dermatological evaluation confirmed the diagnosis of psoriasis vulgaris in the form of patches and plaques, which was further validated histopathologically. Following localized dermatological treatment, her condition improved, with a reduction in the thick scales and gradual clearance of the psoriasis. This case was notable due to the uncommon occurrence of psoriasis vulgaris at such a young age.

Oral administration of acitretin may provide therapeutic benefit in managing Mal de Meleda (MDM); however, its prolonged use is generally discouraged due to the potential for adverse effects. In certain cases, surgical intervention has also been documented as a treatment approach (30).

In summary, establishing a definitive diagnosis of MDM remains a clinical challenge. Evidence suggests that interleukin-17A (IL-17A) may contribute to the inflammatory cascade associated with MDM. High-dose administration of ixekizumab, an IL-17A inhibitor, may offer symptomatic relief in affected individuals. Nevertheless, our study did not assess the expression levels of IL-17A or TNF-α within the lesional tissue prior to therapeutic intervention.

Differentiating MDM from psoriasis is critical due to their distinct therapeutic responses, particularly with respect to biologic agents, as well as differing risks for the development of cutaneous malignancies. Certain clinical indicators, such as age at disease onset and family history, can assist in diagnosis: MDM typically emerges in early infancy and is inherited in an autosomal recessive pattern. Although both MDM and psoriasis may present with sharply defined erythematous plaques with scaling and nail abnormalities like subungual hyperkeratosis and onycholysis, MDM is notably characterized by a transgradient (stocking-and-glove) distribution. Furthermore, features such as sclerodactyly and digital constriction bands (pseudoainhum), which are hallmark signs of MDM, are rarely encountered in psoriasis.

Histopathological differentiation between the two disorders can be difficult, as both conditions may exhibit overlapping microscopic findings, including parakeratosis, orthokeratosis, epidermal thickening (acanthosis), Munro’s microabscesses, and a perivascular lymphocytic infiltrate. However, SLURP-1 immunohistochemistry provides valuable diagnostic insight: in psoriasis, SLURP-1 mRNA expression is upregulated in response to IL-22, whereas in MDM, SLURP-1 is either absent or minimally expressed, facilitating differentiation between the two conditions.

Bertrand Favre and collaborators have shown that SLURP1 functions as a late-stage marker of epidermal differentiation and can be detected in multiple biological secretions, including sweat, saliva, tears, and urine. In the context of Mal de Meleda (MDM), they discovered a novel point mutation—R71H—in the *SLURP1* gene as the pathogenic variant responsible for the disorder. Furthermore, their research was the first to evaluate the functional consequences of several SLURP1 mutations at the protein level. The results demonstrated that SLURP1 expression is markedly reduced or virtually absent in the skin and sweat of individuals affected by MDM. As a result, a straightforward immunological assay may be sufficient to diagnose the majority of MDM cases.

Other hereditary form of palmoplantar keratoderma especially those associated with mutations in KRT1, KRT9 and KRT0, may also present with overlapping clinical features. Although genetic confirmation was not available in this case, the clinical phenotype was highly suggestive of Mal de Meleda. Nonetheless, differential diagnosis should consider such variants, as discussed in recent literature (31).

## Conclusion

In conclusion, it is essential for dermatologists to accurately differentiate Mal de Meleda (MDM) from psoriasis, as both conditions can exhibit overlapping clinical features, yet differ markedly in their therapeutic response and long-term prognosis. Distinctive characteristics that aid in distinguishing MDM include its very early onset, autosomal recessive mode of inheritance, and the presence of a transgradient pattern of palmoplantar keratoderma, features that are not typically observed in psoriasis vulgaris. Although biologic agents targeting tumor necrosis factor-alpha (TNF-α) and interleukin-17A (IL-17A) have demonstrated limited therapeutic benefit in MDM, systemic retinoids continue to offer a practical treatment avenue. The clinical case presented here was notable for its early manifestation and a familial history of similar dermatoses.

Beyond the physical manifestations, psoriasis imposes a considerable emotional and psychosocial burden, affecting patients’ social interactions and interpersonal relationships. Acknowledging the psychological and psychiatric comorbidities associated with psoriasis is critical to comprehensive care. Tailored interventions—such as cognitive-behavioral therapies, supportive counseling, and pharmacological treatment for mood disorders—can enhance disease management and improve overall quality of life. Further studies are warranted to investigate psychological traits, including temperament and attachment styles, among individuals with chronic dermatological conditions, which may inform more personalized therapeutic strategies.

To summarize, clear differentiation between MDM and psoriasis remains imperative, given their contrasting therapeutic pathways and disease trajectories. Hallmarks such as neonatal or early childhood onset, hereditary transmission via autosomal recessive inheritance, and characteristic transgradient keratoderma are pivotal for recognizing MDM. While standard biologic therapies used in psoriasis offer limited benefit in MDM, retinoid-based regimens and topical keratolytics are central to its management.

Furthermore, while the cytokine milieu comprising TNF-α, IL-17, and IL-22 has been extensively delineated in psoriasis pathophysiology, its specific role in MDM remains inadequately characterized. Our observations reaffirm that biologics directed against TNF-α and IL-17A show minimal clinical response in MDM cases. Consequently, systemic retinoids and keratolytic topical agents remain the cornerstone of treatment, as conventional immunosuppressive therapies used in psoriasis—such as methotrexate and cyclosporine—have limited utility in the context of MDM.

## Data Availability

The original contributions presented in this study are included in this article/supplementary material, further inquiries can be directed to the corresponding author.
